# Supplementary figures and images for: Optogenetic Tools for Control of Public Goods in Saccharomyces cerevisiae
Source: mSphere. 2021 Aug 25;6(4):e00581-21. doi: 10.1128/mSphere.00581-21 (PMC8386412; doi:10.1128/mSphere.00581-21)

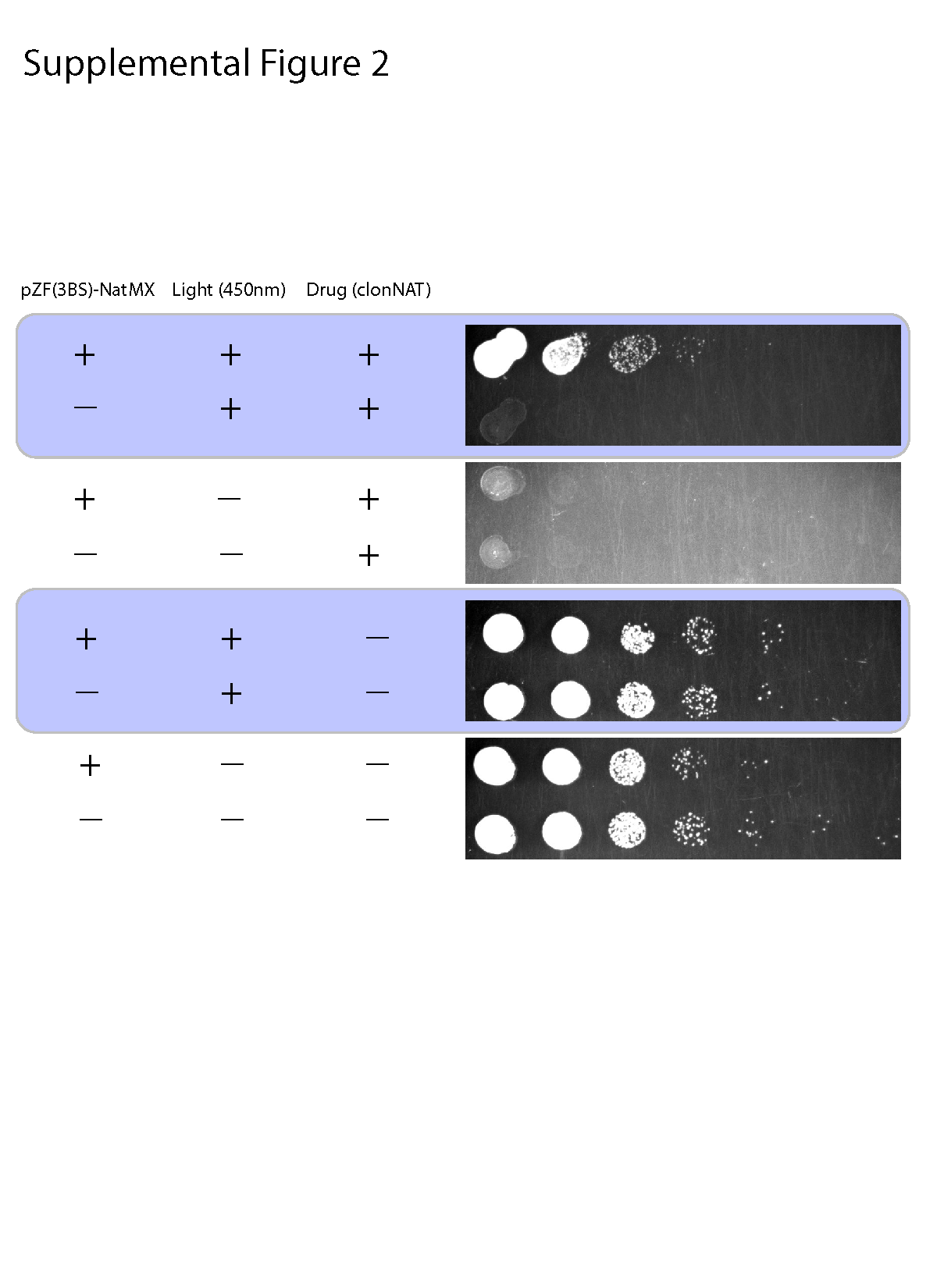

Supplement: FIG S2 [file msphere.00581-21-sf002.tif]

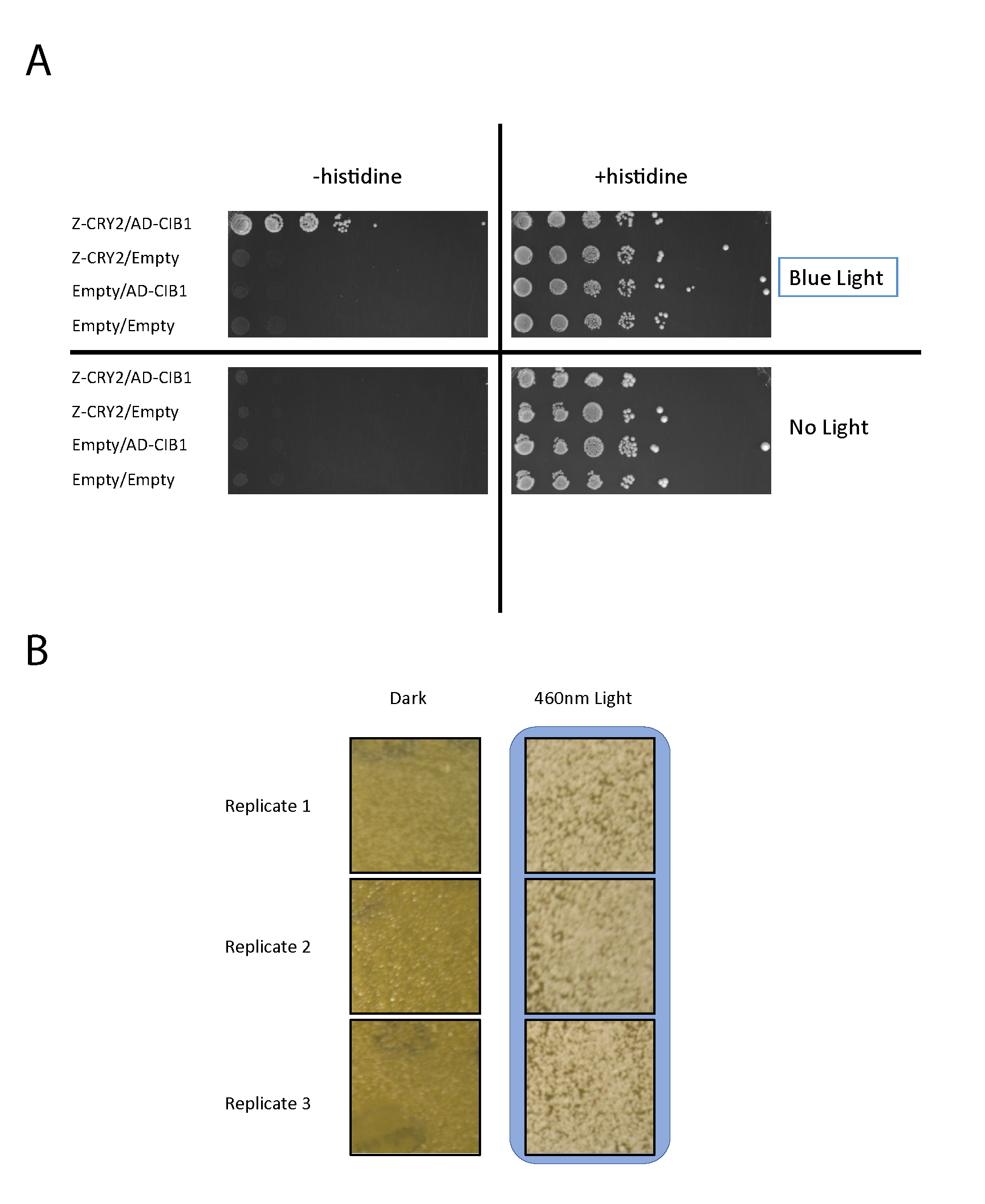

Supplement: FIG S5 [file msphere.00581-21-sf005.tif]

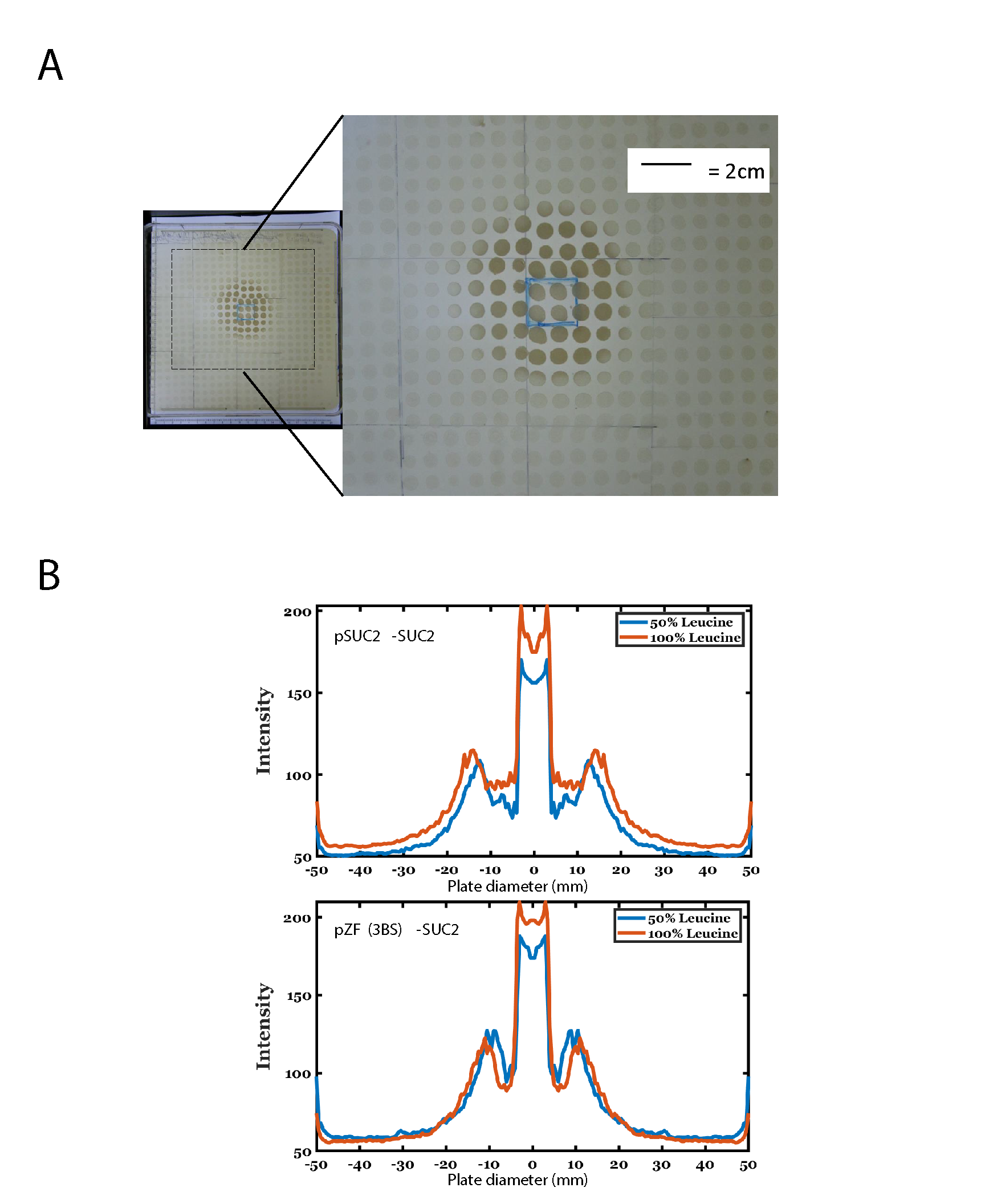

Supplement: FIG S7 [file msphere.00581-21-sf007.tif]

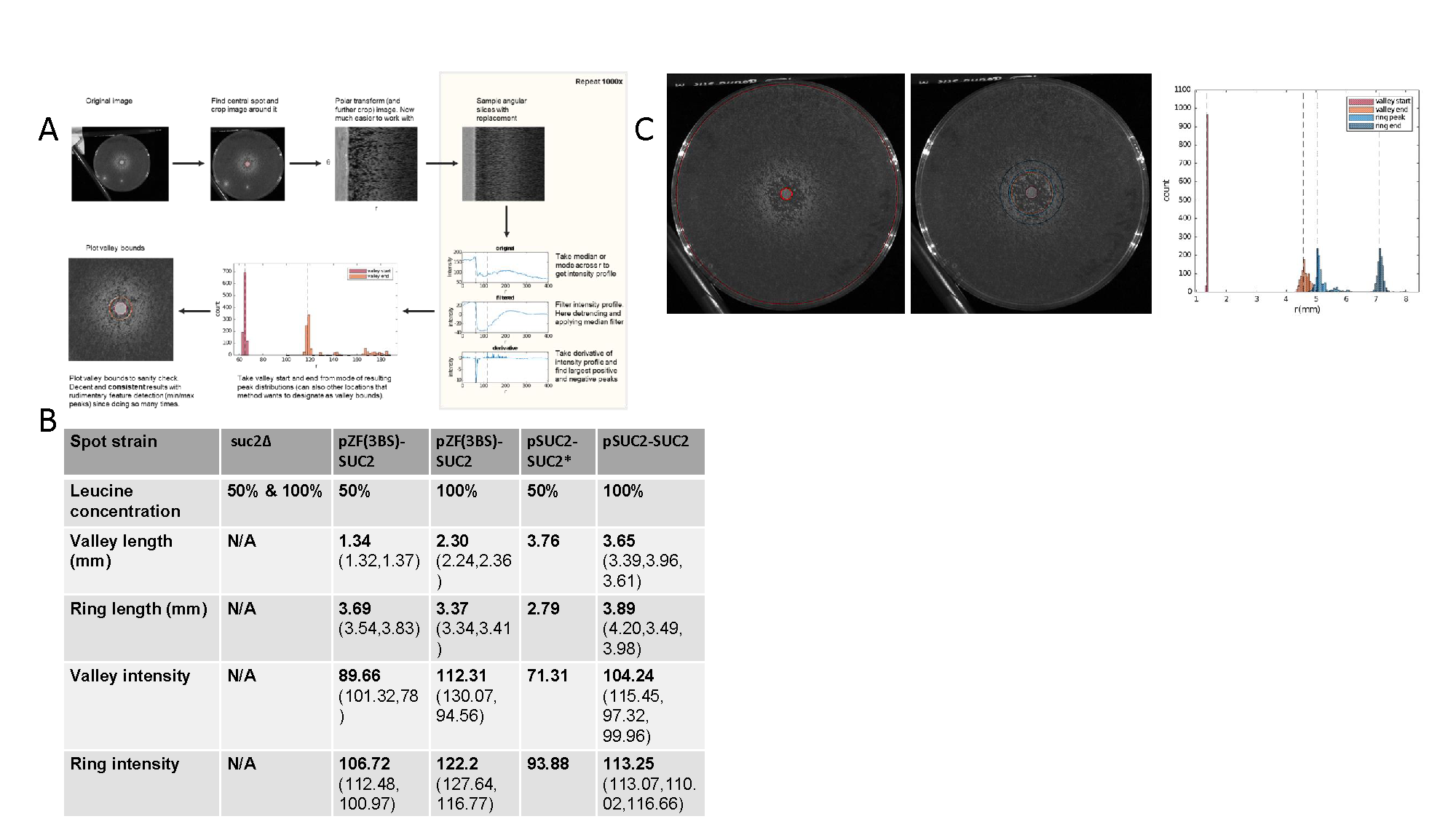

Supplement: FIG S8 [file msphere.00581-21-sf008.tif]
