# Supplementary material for: Optogenetic Tools for Control of Public Goods in Saccharomyces cerevisiae
Source: mSphere. 2021 Aug 25;6(4):e00581-21. doi: 10.1128/mSphere.00581-21 (PMC8386412; doi:10.1128/mSphere.00581-21)
Supplement: TEXT S1 [file msphere.00581-21-t0001.docx]

**Supplemental Material**

**Optogenetic tools for public goods control in *Saccharomyces cerevisiae***

Neydis Moreno Morales^1^, Michael T. Patel^2^, Cameron J. Stewart^1^ , Kieran Sweeney^1^ and Megan. N. McClean^1ǂ^

**AFFILIATIONS**

1. Department of Biomedical Engineering, University of Wisconsin-Madison, Madison, WI USA
2. Lewis-Sigler Institute for Integrated Genomics, Princeton University, Princeton, NJ USA

ǂ Corresponding author email: mmcclean@wisc.edu

Contents

[Supplemental Methods 3](#_Toc78446683)

[Plasmid Construction 3](#_Toc78446684)

[Strain Construction 4](#_Toc78446685)

[References 4](#_Toc78446686)

# Supplemental Methods

### Plasmid Construction

The plasmid carrying GAL4DBD-CRY2 loxP-KIURA3-loxP CIB1-VP16 (pMM335) was created using yeast homologous recombination into pMM7. The GAL4AD-CIB1 cassette was PCR amplified using primers oMM628 and oMM629. The primer oMM628 contains (from 5’ to 3’) homology to pMM7, an added XmaI site, and forward priming to pMM159. The primer oMM629 contains (5’ to 3’) homology to loxP-KLURA3-loxP cassette and reverse priming to pMM159. The loxP-KLURA3-loxP cassette was PCR amplified from pMM326 with primers oMM250 and oMM251. The GAL4BD-CRY2 cassette was PCR amplified with primers oMM630 and oMM631. The primer oMM630 consists of homology to the downstream region of loxP-KLURA3-loxP and forward priming to pMM160. The primer oMM631 consists of homology to pMM7, an added PacI site, and reverse priming to pMM160. The ADH1 promoters and terminators were preserved for both CIB1 and CRY2 amplifications. XmaI and PacI restriction sites are not present in the final construct except for where they were included by PCR. For yeast homologous recombination, pMM7 was digested to completion at its unique SacI site and cotransformed with the three PCR products listed above into yMM1146 and plated on SC –URA-LEU for selection. Colonies were picked and plasmid recovered with the Zymo Yeast Plasmid Miniprep Kit. Plasmid verified by digest with HindIII and sequencing. To generate an integrating version (pMM337), the cassette from pMM335 containing GAL4DBD-CRY2 loxP-KIURA3-loxP VP16-CIB1 was cloned into pMM327 between the XmaI and PacI cassettes.

The plasmid containing ZDBD-CRY2PHR loxP-KIURA3-loxP CIB1-VP16 was generated using the same scheme as for pMM335 with the following exceptions: oMM653 replaced oMM630 to amplify the ZDBD-CRY2PHR construct, oMM664 replaced oMM628 and contains a PacI site instead of XmaI, and oMM665 replaces oMM631 and contains an AscI site instead of PacI. To increase the spacing between the ZDBD-CRY2PHR and CIB1-VP16 the resulting plasmid was linearized using BsiWI and a fragment containing the tetR sequence from pBR322 (amplified with oMM681/682) was ligated between CIB1-VP16 and the loxP-URA3-loxP cassette. The ZDBD-CRY2 loxP-KIURA3-loxP Spacer CIB1-VP16 cassette from the resulting plasmid (pMM364) was liberated using PacI/AscI and ligated into pMM327 to generate a plasmid capable of integration at the HO locus. The plasmid (pMM359) containing Gal4AD-CIB1 loxP-KIURA3-loxP ZDBD-CRY2PHR was generated using the same scheme as for pMM364. This plasmid was then cut and ligated into pMM327 as described for pMM364 and integrated into yMM1146 to make yMM1355 which was used with pMM369 (yMM1377) and pMM6 (yMM1378) to demonstrate light-induced drug resistance in **Supplemental Figure 2.**

The pGAL1-yEVENUS episomal reporter on a scTRP1 backbone (pMM336) was constructed by cutting pMM301 with PvuII and ligating into the corresponding site on pMM6.

The pZF(3BS)-yEVENUS scTRP1 CEN reporter plasmid (pMM365) was created using yeast homologous recombination. The plasmid pMM287 (pZF(3BS)-yEVENUS scURA3 CEN) was digested with ApaI and co-transformed with TRP1 amplified from pMM6 using oMM611/612 into yMM1146, selected on SC-TRP and recovered by plasmid prep and transformation into *E. coli* competent cells.

The pZF(3BS)-NatMX construct was constructed using yeast recombinational cloning by amplifying the NatMX cassette from pMM129 with oMM687/688 and co-transforming with pMM365 cut with EcoIR and AscI into yMM1146 and selecting for TRP+ yeast. Plasmids were prepped from yeast, transformed into *E. coli*, and verified by sequencing.

### Strain Construction

The integrated pZF(3BS)-mRUBY2 strain was generated by transforming the unrecycled optogenetic strain (yMM1367 Matα trp1∆63 leu2∆1 ura3-52 HO::SV40NLS-VP16-CIB1 loxP-KLURA3-loxP SV40NLS-Zif268DBD-CRY2PHR) with pMM553 linearized at NotI to generate yMM1427.

The pZF(4BS)-HIS3 strain was created by swapping pGAL1-HIS3 with pZF(4BS)-HIS3 in a common yeast two-hybrid strain [3] using primers oMM575/576 to amplify KanMX4-pZF(4BS) from pMM299 and transform into yMM770. Strains were selected for G418 resistance and checked with oMM624, 625.

The pZF(4BS)-yVENUS reporter was created by yeast recombinational cloning by digesting pMM299 with EcoRI, amplifying yEVENUS from pMM289 with oMM191/626 and co-transforming into yMM1146. Plasmids were recovered from yeast and sequenced before using with pMM159/pMM284 to assess expression from the pZF(4BS) promoter.

The pZF(3BS)-yEVENUS strain was created by linearizing pMM287 at URA, removing the CEN/ARS and integrating into yMM1146 and selecting for URA+ transformants.

Initial tests of the system were performed using strains (yMM1332, 1351) with integrated version of the GAL4DBD-CRY2/Gal4AD-CIB1 split transcription factor. Strain yMM1351 (Matα trp1∆63 leu2∆1 ura3-52 pADH1-GAL4AD-CIB1-tADH2 loxP-KIURA3-loxP pADH1-GAL4BD-CRY2-tADH2) was generated by integrated pMM337 at the HO and loxing out the KIURA3 marker using pMM296 as described in the main text to generate yMM1332 (Matα trp1∆63 leu2∆1 ura3-52 pADH1-GAL4AD-CIB1-tADH2 loxP pADH1-GAL4BD-CRY2-tADH2).

Strain yMM1367 (Matα trp1∆63 leu2∆1 ura3-52 HO::SV40NLS-VP16-CIB1 loxP-kKlURA30loxP SV40NLS-Zif268DBD-CRY2PHR) was constructed by ligating the SV40NLS-VP16-CIB1 loxP-KlURA3-loxP SV40NLS-Zif268DBD-CRY2PHR cassette from pMM364 into pMM327, linearizing the resulting plasmid with AatII and integrating at the HO locus. Appropriate integration was checked by colony PCR and by the ability of the resulting strain to induce blue-light expression of yEVENUS when transformed with pMM365 (pZF(3BS)-yEVENUS). To generate yMM1390 (Matα trp1∆63 leu2∆1 ura3-52 HO::SV40NLS-VP16-CIB1 loxP SV40NLS-Zif268DBD-CRY2PHR) we used Cre-recombinase mediated recycling to remove the KlURA3 marker following the protocol described in the main text. The light inducible SUC2 strain (yMM1406) was constructed by transforming yMM1390 with the PCR product of pMM353 and oMM768/769 consisting of the KanMX4-pZF(3BS) cassette amplified with appropriate homology to replace the pSUC2 promoter with pZF(3BS). Transformants were checked by colony PCR and the ability to grow on YP-Sucrose only in blue-light.

# References

| [1] | E. Andersen, "tPCR-Directed In Vivo Plasmid Construction Using Homologous Recombination in Baker’s Yeast," *Molecular Methods for Evolutionary Genetics. Methods in Molecular Biology ,* vol. 772, pp. 409-421, 8 7 2011. |
| --- | --- |
| [2] | R. McIsaac, S. Silverman, M. McClean, P. Gibney, J. Macinskas, M. Hickman, A. Petti and a. D. Botstein, "Fast-acting and nearly gratuitous induction of gene expression and protein depletion in Saccharomyces cerevisiae.," *Molecular Biology of the Cell,* vol. 22, pp. 4447-4459, 2011. |
| [3] | J. An-Adirekkun, C. Stewart, S. Geller, M. Patel, J. Melendez, B. Oakes, M. Noyes and M. McClean, "A yeast optogenetic toolkit (yOTK) for gene expression control in Saccharomyces cerevisiae," *Biotechnology and Bioengineering,* vol. 117, no. 3, pp. 886-893, 2020. |
| [4] | R. Sikorski and P. Hieter, "A system of shuttle vectors and yeast host strains designed for efficient manipulation of DNA in Saccharomyces cerevisiae," *Genetics,* vol. 122, no. 1, pp. 19-27, 1989. |
| [5] | U. Gueldener, J. Heinisch, G. Koehler, D. Voss and J. Hegemann, "A second set of loxP marker cassettes for Cre-mediated multiple gene knockouts in budding yeast," *Nucleic acids research,* vol. 30, no. 6, 2002. |
| [6] | W. Voth, J. Richards, J. Shaw and D. Stillman, "Yeast vectors for integration at the HO locus," *Nucleic Acids Research,* vol. 29, no. 12, p. e59, 2001. |
