# Supplementary material for: Optogenetic Tools for Control of Public Goods in Saccharomyces cerevisiae
Source: mSphere. 2021 Aug 25;6(4):e00581-21. doi: 10.1128/mSphere.00581-21 (PMC8386412; doi:10.1128/mSphere.00581-21)
Supplement: TABLE S1 [file msphere.00581-21-st001.docx]

### Supplemental Table 1: Supplemental Biological Materials

| ID | Alias | Type | Genotype/Sequence/Genes | Target | Notes | Source |
| --- | --- | --- | --- | --- | --- | --- |
| yMM0770 | Yeast2Hybrid | Yeast | MATa trp1-901 leu2-3,112 ura3-52 his3-200 gal4Δ gal80Δ LYS2::GAL1-HIS3 GAL2-ADE2 met2::GAL7-lacZ |  | Yeast two-hybrid strain (HIS3 expression) | James , et al 1996 [3] |
| yMM1146 | DBY8750, KSY1284 | Yeast | Matα trp1∆63 leu2∆1 ura3-52 |  |  | Botstein lab |
| yMM1295 | pZF(4BS)-scHIS3 | Yeast | MATa trp1-901 leu2-3,112 ura3-52 his3-200 gal4Δ gal80Δ LYS2::KanMX4-pZF(4BS)-HIS3 GAL2-ADE2 met2::GAL7-lacZ |  | HIS3 expression under pZF(4BS) | This Study |
| yMM1332 | GAL4AD-CIB1 GAL4BD-CRY2 | Yeast | Matα trp1∆63 leu2∆1 ura3-52 pADH1-GAL4AD-CIB1-tADH2 loxP pADH1-GAL4BD-CRY2-tADH2 |  | Strain with integrated GAL4DBD -CRY2/CIB1-AD optogenetic system. Marker (KIURA3) has been lox’d out. | This Study |
| yMM1351 | yMM1146 CIB1-KLURA3-CRY2 | Yeast | Matα trp1∆63 leu2∆1 ura3-52 pADH1-GAL4AD-CIB1-tADH2 loxP-KIURA3-loxP pADH1-GAL4BD-CRY2-tADH2 |  | Strain with integrated GAL4DBD -CRY2/CIB1-AD optogenetic system. Marker (KIURA3) has not been lox’d out. | This Study |
| yMM1353 | yMM1146_pMM287 | Yeast | Matα trp1∆63 leu2∆1 URA3::pZF(3BS)-yEVenus |  | Yeast | This Study |
| yMM1355 |  | Yeast | Matα trp1∆63 leu2∆1 ura3-52 HO::GAL4AD-CIB1 loxP-KLURA3-loxP FLAG(3X)-SV40NLS- Zif268DBD -CRYPHR |  |  | This Study |
| yMM1367 | yMM1146_pMM364atHO | Yeast | Matα trp1∆63 leu2∆1 ura3-52 HO::SV40NLS-VP16-CIB1 loxP-KLURA3-loxP SV40NLS-Zif268DBD-CRY2PHR |  | Strain with integrated optogenetic system, to allow for induction of GOI using KanMXREV-pZF promoter | An-Adirekkun, et al 2020 [3] |
| yMM1377 | yMM1377+pMM369 | Yeast | Matα trp1∆63 leu2∆1 ura3-52 HO::GAL4AD-CIB1 loxP-KLURA3-loxP FLAG(3X)-SV40NLS- Zif268DBD -CRYPHR [pMM369] |  |  | This Study |
| yMM1378 | yMM1377+pMM6 | Yeast | Matα trp1∆63 leu2∆1 ura3-52 HO::GAL4AD-CIB1 loxP-KLURA3-loxP FLAG(3X)-SV40NLS- Zif268DBD -CRYPHR [pMM6] |  |  | This Study |
| yMM1390 | yCS16 | Yeast | Matα trp1∆63 leu2∆1 ura3-52 HO::SV40NLS-VP16-CIB1 loxP SV40NLS-Zif268DBD-CRY2PHR |  | Yeast | This Study |
| yMM1406 | pZF-SUC2, yCS22 | Yeast | Matα trp1∆63 leu2∆1 ura3-52 HO::SV40NLS-VP16-CIB1 loxP SV40NLS-Zif268DBD-CRY2PHR KanMX-pZF(3BS)-SUC2 |  | Yeast | This Study |
| yMM1427 | pZF-mRUBY2 | Yeast | Matα trp1∆63 leu2∆1 ura3-52 HO::SV40NLS-VP16-CIB1 loxP-KLURA3-loxP SV40NLS-Zif268DBD-CRY2PHR |  |  | This Study |
| oMM0166 | rev_pGal4AD-CIB1_check | Oligo | aagtgaacttgcggggtttt | CIB1 | Colony PCR |  |
| oMM0191 | Forward_pGAL1 | Oligo | GCGAAGCGATGATTTTTGAT |  |  |  |
| oMM0250 | Lox_General_for | Oligo | cgtacgctgcaggtcgac | loxP cassette | Amplification |  |
| oMM0251 | Lox_General_rev | Oligo | cactatagggagaccggcag | loxP cassette | Amplification |  |
| oMM0575 | revKanMX_pGAL_4xZif_reverse_YRC | Oligo | GTCGACGGTATCGATAAGCTTGATATCGAATTCCTGCAGCatagttttttctccttgacg | KanMX | Amplification & Transformation |  |
| oMM0576 | revKANMX_LYS2_forward | Oligo | TTCAGGTGCTGGTGCTCGTGGAAGCTCCGCAGCAGCTTAAcgcacttaacttcgcatctg | KanMX | Amplification & Transformation |  |
| oMM0611 | pRS_markerswap_for | Oligo | CTTAACTATGCGGCATCAGA | pRS marker | Yeast recombinational cloning |  |
| oMM0612 | pRS_markerwap_rev | Oligo | CCTGATGCGGTATTTTCTCC | pRS marker | Yeast recombinational cloning |  |
| oMM0624 | LYS2_forward | Oligo | CTAGTTGCTTCAGGTGCTGG | LYS2 | Colony PCR/checking |  |
| oMM0625 | HIS3_reverse | Oligo | GCCTGTTCTGCTACTGCTTC | HIS3 | Colony PCR/checking |  |
| oMM0626 | Venus_reverse | Oligo | CCAGTGAGCGCGCGTAATACGACTCACTATAGGGCGAATTaggaaacagctatgaccatg | Venus | Yeast recombinational cloning |  |
| oMM0628 | pRS_XmaI_CIB1_forward | Oligo | GTCGACGGTATCGATAAGCTTGATATCGAATTCCcccggggatggtggtacataacgaac | pRS vector | Yeast recombinational cloning |  |
| oMM0629 | loxP_CIB1_reverse | Oligo | ACGAAGTTATATTAAGGGTTGTCGACCTGCAGCGTACGtggaatatgttcatagggtagg | CIB1/loxP cassette | Yeast recombinational cloning |  |
| oMM0630 | loxP_CRY2_forward | Oligos | AGTTATTAGGTGATATCAGATCCACTAGTGGCCTATGCGGtccctaacatgtaggtggcg | loxP cassete/CRy2 | Yeast recombinational cloning |  |
| oMM0631 | CRY2_pRS_PacI_reverse | Oligos | GATTACGCCAAGCGCGCAATTAACCCTCACTATTAATTAAcatgccggtagaggtgtggt | CRY2 | Yeast recombinational cloning |  |
| oMM0646 | HO-L forward_check | Oligos | GGGAGAACGAGTACCTGTAG | HO | Colony PCR |  |
| oMM0647 | CIB1 reverse_check | Oligos | CTTTGCAAAGCTTGGAGTTG | CIB1 | Colony PCR |  |
| oMM0650 | tADH2_downstream_forward_seq | Oligos | CAGGTATAGCATGAGGTCGC | tADH1 | Sequencing/Colony PCR |  |
| oMM0651 | pMM160_upstream_reverse_seq | Oligos | CCACCATCAATGAGGCAGTG | pADH1 | Sequencing/Colony PCR |  |
| oMM0652 | CIB1_downstream_for_seq | Oligos | GGCCATGTAACCTCTGATCT | CIB1 | Sequencing/Colony PCR |  |
| oMM0653 | loxP_ZCRY2_forward | Oligos | AGTTATTAGGTGATATCAGATCCACTAGTGGCCTATGCGGcgatttagagcttgacgggg | ZCRY2 | Yeast Recombinational Cloning |  |
| oMM0664 | pRS_PacI_CIB1_Forward | Oligos | GTCGACGGTATCGATAAGCTTGATATCGAATTTTAATTAAgatggtggtacataacgaac | CIB1 | Yeast Recombinational Cloning |  |
| oMM0665 | CRY2-AscI-pRS Reverse | Oligo | GATTACGCCAAGCGCGCAATTAACCCTCACTAGGCGCGCCcatgccggtagaggtgtggt | CRY2 | Yeast Recombinational Cloning |  |
| oMM0673 | ZF(3BS)_for | Oligo | CTAGACGCCCACGCTCGCCCACGCTCGCCCACGCGC | pMM347 ZFBS | Anneal and ligate |  |
| oMM0674 | ZF(3BS)_rev | Oligo | GGCCGCGCGTGGGCGAGCGTGGGCGAGCGTGGGCGT | pMM347 ZFBS | Anneal and ligate |  |
| oMM0675 | ZF(2BS)_for | Oligo | CTAGACGCCCACGCCGCCCACGCCCACGCGC | pMM347 ZFBS | Anneal and ligate |  |
| oMM0676 | ZF(2BS)_rev | Oligo | GGCCGCGCGTGGGCGTGGGCGGCGTGGGCGT | pMM347 ZFBS | Anneal and ligate |  |
| oMM0681 | BsiWI_tetR_for | Oligo | ggtcgtacgGCGCTCATCGTCATCCTCGG | tetR | Cloning |  |
| oMM0682 | BsiWI_tetR_rev | Oligo | cctcgtacgCTAGCCGGGTCCTCAACGAC | tetR | Cloning |  |
| oMM0684 | KLURA3_rev | Oligo | GAATCAGCGCTCCCCATTAA | KIURA3 | Sequencing/Colony PCR |  |
| oMM0687 | NatMX_for | Oligo | cctctatactttaacgtcaaggagaaaaaactataaccactcttgacgacacggcttacc | NatMX | Yeast recombinational cloning |  |
| oMM0688 | NatMX_rev | Oligo | atcataaatcataagaaattcgcttatttagaagtggggcagggcatgctcatgtagagc | NatMX | Yeast recombinational cloning |  |
| oMM0745 | Lox_check_for | Oligo | GTCATGCTTCAACAATGGCGA | loxP | Checking/colony PCR |  |
| oMM0746 | Lox_check_rev | Oligo | TAAGCTCATCCGAGCGAGAAA | loxP | Checking/colony PCR |  |
| oMM0768 | scSUC2_pZF_forward | Oligo | CTCAGAGAAACAAGCAAAACAAAAAGCTTTTCTTTTCACTaacaaaagctggagctgcat | SUC2 promoter | Amplification & Transformation |  |
| oMM0769 | scSUC2_pZF_rev | Oligo | CAAAAGGAAAAGGAAAGCTTGCAAAAGCATCATATACGTTattgggacaacaccagtgaa | SUC2 promoter | Amplification & Transformation |  |
| pBR322 | pBR322 | Plasmid | N/A AMP^R^ |  |  | Gift from Rose/Gammie Lab |
| pMM0006 | pRS414 | Plasmid | scTRP1 CEN6 ARS4 AMP^R^ |  |  | Sikorski and Hieter, 1989 [4] |
| pMM0007 | pRS415 | Plasmid | scLEU2 CEN6 ARS4 AMP^R^ |  |  | Sikorski and Hieter, 1989 [4] |
| pMM0008 | pRS416 | Plasmid | scURA3 CEN6 ARS4 AMP^R^ |  |  | Sikorski and Hieter, 1989 [4] |
| pMM0129 | NatMX | Plasmid | Nat1 AMP^R^ |  |  | Goldstein and McCusker, 1999 [5] |
| pMM0159 | pGal4AD-CIB1 | Plasmid | pscADH1-GAL4AD-CIB1-tscADH1 AMP^R^ |  |  | Kennedy, et al 2010 [7] |
| pMM0160 | pGal4DBD-CRY2 | Plasmid | pscADH1-GAL4DBD-CRY2-tscADH1 AMP^R^ |  |  | Kennedy, et al 2010 [7] |
| pMM0284 | FLAG(3X)-NLSZIF268DBD-CRY2 (L3) | Plasmid | pscADH1-pFLAG(3X)-NLS-ZIF268DBD-CRY2 (L3)-tscADH1 TRP1 AMP^R^ |  |  | An-Adirekkunet al 2020 [3] |
| pMM0287 | pZF(3BS) URA | Plasmid | pZF(3BS)-yEVENUS CEN scURA3 AMP^R^ |  |  | An-Adirekkunet al 2020 [3] |
| pMM0289 | pZF(4BS) URA | Plasmid | pZF(4BS)-yEVENUS CEN scURA3 AMP^R^ |  |  | An-Adirekkunet al 2020 [3] |
| pMM0296 | pSH65 | Plasmid | pGAL1-CRE PhleoR AMP^R^ |  |  | Botstein Lab; Gueldeneret al 2002 [5] |
| pMM0299 | KanMXrev-pZF(4BS) | Plasmid | KanMXrev-pZF(4BS) in pIDTBlue AMP^R^ |  |  | This Study |
| pMM0301 | pGAL1-yEVENUS | Plasmid | pGAL1-yEVENUS scURA3 CEN AMP^R^ |  |  | An-Adirekkunet al 2020 [3] |
| pMM0326 | pUG72 | Plasmid | loxP-KIURA3-loxP AMP^R^ |  |  | Gueldener*et al* 2002 [5] |
| pMM0327 | HO-Polylinker-HO | Plasmid | HO-Polylinker-HO AMP^R^ |  |  | Voth, et al 2001 [6] |
| pMM0335 | GAL4ADCIB1/GAL4DBDCRY2 | Plasmid | GAL4AD-CIB1 loxP-KIURA3-loxP GAL4DBD-CRY2 LEU2 Cen/ARS AMP^R^ |  |  | This Study |
| pMM0336 | pGAL1-YFP | Plasmid | pGAL1-yEVENUS CEN scTRP1 AMP^R^ |  |  | This Study |
| pMM0337 | HO GAL4 split | Plasmid | HO-GAL4AD-CIB1 loxP-KIURA3-loxP GAL4DBD-CRY2-HO AMP^R^ |  |  | This Study |
| pMM0347 | pMM299-VENUS | Plasmid | KanMXrev-pZF(4BS)-VENUS in pIDTBlue AMP^R^ |  |  | This Study |
| pMM0353 | KanMXrev-pZF(3BS) | Plasmid | KanMXrev-pZF(3BS)-VENUS in pIDTBlue AMP^R^ |  |  | This Study |
| pMM0354 | KanMXrev-pZF(2BS) | Plasmid | KanMXrev-pZF(2BS)-VENUS in pIDTBlue AMP^R^ |  |  | This Study |
| pMM0359 | Gal4AD-CIB1/ZCRY2-PHR HO | Plasmid | GAL4AD-CIB1 loxP-KLURA3-loxP FLAG(3X)-SV40NLS-Zif-ZCRYPHR LEU2 CEN/ARS AMP^R^ |  |  | This Study |
| pMM0364 | CIB1/CRY2PHR loxURA CEN | Plasmid | SV40NLS-VP16-CIB1 loxP-KLURA3-loxP SV40NLS-ZIF268DBDCRY2PHR LEU2 CEN/ARS AMP^R^ |  |  | This Study |
| pMM0365 | pZF(3BS) TRP1 | Plasmid | pZF(3BS)-yEVENUS CEN scTRP1 AMP^R^ |  |  | This Study |
| pMM0369 | pZF(3BS)-NatMX | Plasmid | pZF(3BS)-NatMX scTRP1 CEN AMP^R^ |  |  | This Study |
| pMM0553 | pZF(mRUBY2) @LEU2 | Plasmid | Leu2 5’ homology-pZF(3BS)-mRUBY2-tADH1-Con1-LEU2-Leu2 3’ homology AMP^R^ |  |  | An-Adirekkunet al 2020 [3] |
